# Necrotizing Fasciitis of the Serratus Anterior in a Patient Treated With Infliximab and Prednisolone for Ulcerative Colitis and Rheumatoid Arthritis

**DOI:** 10.7759/cureus.59346

**Published:** 2024-04-30

**Authors:** Natsuki Okai, Yasuo Otsuka, Sho Masaki, Masatoshi Kudo, Tomohiro Watanabe

**Affiliations:** 1 Gastroenterology and Hepatology, Kindai University Faculty of Medicine, Osaka-Sayama, JPN

**Keywords:** group a streptococcus, purpura fulminans, prednisolone, infliximab, necrotizing fasciitis

## Abstract

Necrotizing fasciitis (NF) is a rapidly progressive bacterial infection with high mortality. Invasive group A *Streptococcus* (GAS) infection is the leading cause of NF. Our understanding regarding clinicopathological features and pathogenesis of invasive GAS infection is expanding as the incidence of NF in healthy individuals increases. However, clinicopathological features of NF in the presence of autoimmune diseases have been poorly defined. We experienced NF in a patient treated with infliximab and prednisolone for ulcerative colitis and rheumatoid arthritis. Herein, we present time kinetics findings of clinical symptoms and laboratory data of GAS-associated NF in the presence of immunosuppressant-treated immune disorders.

## Introduction

Although group A Streptococcus (GAS, *Streptococcus pyogenes*) usually causes innocuous infections such as pharyngitis and impetigo, much attention has been paid to this organism since the discovery of necrotizing fasciitis (NF, also known as the flesh-eating disease) and streptococcal toxic shock syndrome caused by invasive infection with GAS [1-3]. NF is a rapidly progressive bacterial infection involving the skin, subcutaneous and deep soft tissues, and muscle [1-3]. The mortality of GAS-associated NF exceeds 30-50% and therefore rapid diagnosis followed by prompt surgical debridement and aggressive antibiotics therapy is required [1,2].

Invasive infection with GAS is increasing in multiple countries in the northern and southern hemispheres including European countries, United States, Japan, and Australia [4-6]. Thus, cases of invasive GAS infection are increasing in the developed countries. In parallel to such a surge of invasive GAS infection, our understanding of clinical features and pathogenesis of NF is expanding [1,2]. On the other hand, it remains largely unknown whether patients treated with immunosuppressants including biologics and steroids are vulnerable to invasive GAS infection. Moreover, few case reports of NF have been reported in the setting of autoimmune disorders treated with immunosuppressants. Here we report a case with NF of the left serratus anterior due to invasive GAS infection in a patient treated with infliximab (IFX) and prednisolone (PSL) for ulcerative colitis (UC) and rheumatoid arthritis (RA).

## Case presentation

A 68-year-old woman complained of sudden onset pain in the left lateral chest wall. She had been treated with IFX (5 mg/kg) every eight weeks in addition to 5-aminosalicylic acid (4 g/day) for UC for approximately 10 years. She was also treated with PSL (7.5 mg/day) for RA. Her UC and RA had been in the remission stage for approximately five years. She did not complain of a sore throat. On admission, no trauma, injury, or rashes were seen on her chest wall and her vital signs were normal. Laboratory findings are shown in Table 1.

**Table 1:** Changes in laboratory examinations ND, not determined.

Hours after admission (hr)	0	10	24	Normal range
Complete blood count				
White blood cell counts (/µL)	10,120	8,330	13,110	3300-8600
Red blood cell counts (x10^4^/µL)	387	360	390	386-492
Hemoglobin (g/dL)	11.5	10.8	11.7	11.6-14.8
Hematocrit (%)	35.0	32.6	36.7	35.1-44.4
Platelet counts (x10^4^/µL)	8.5	9.6	12.2	15.8-34.8
Coagulation				
Prothrombin time (%)	107.7	81.2	46.3	70-130
Activated partial thromboplastin time (sec)	32.5	35.9	43.2	24-39
D-dimer (µg/mL)	2.1	3.2	6.8	0-1
Fibrinogen (mg/dL)	ND	ND	597	200-400
Biochemistry				
Total protein (mg/dL)	6.4	5.2	5.1	6.6-8.1
Albumin (mg/dL)	3.5	2.7	2.5	4.1-5.1
Total bilirubin (mg/dL)	1.0	1.0	1.0	0.4-1.5
Amylase (U/L)	60	70	82	44-132
Aspartate aminotransferase (U/L)	46	49	89	13-30
Alanine aminotransferase (U/L)	63	46	66	7-23
$\gamma$-glutamyl transpeptidase (U/L)	101	77	72	9-32
Lactate dehydrogenase (U/L)	234	226	354	124-222
Creatine phosphokinase (U/L)	54	1144	2676	41-153
Creatine phosphokinase-MB (U/L)	1	12	26	0-13
Blood urea nitrogen (mg/dL)	22	29	33	8-20
Creatinine (mg/dL)	0.83	1.44	1.76	0.46-0.79
Na (mmol/L)	141	142	144	138-145
K (mmol/L)	3.6	3.2	4.3	3.6-4.8
Cl (mmol/L)	103	107	109	101-108
Glucose (mg/dL)	123	126	184	73-109
Inflammation				
C-reactive protein (mg/dL)	2.561	16.112	35.237	0-0.14
Procalcitonin (ng/mL)	1.3	73.50	110.19	0-0.5
IL-6 (pg/mL)	ND	6697	ND	0-7

**Figure 1:**
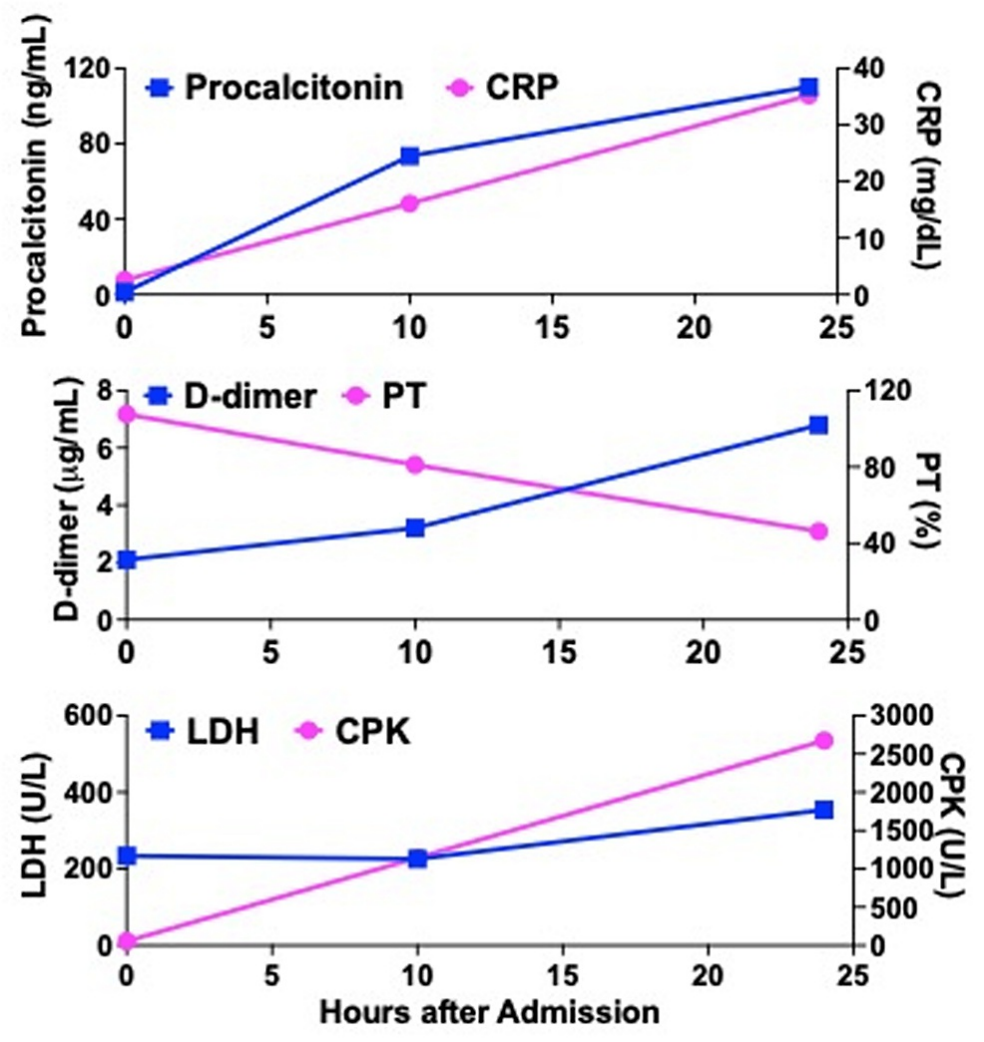
Kinetics of serum inflammatory, coagulation, and biochemical parameters after admission. CPK, creatine phosphokinase, CRP, C-reactive protein, LDH, lactate dehydrogenase, PT, prothrombin time

Blood examinations showed mild elevation of white blood cell (WBC) counts and thrombocytopenia. Mild elevation in serum levels of C-reactive protein (CRP, Table 1 and Figure 1), procalcitonin, and D-dimer were also seen. Additionally, serum levels of hepatobiliary enzymes and lactate dehydrogenase were elevated. Other biochemical and coagulation parameters were within normal limits. We could not reach the diagnosis accounting for her lateral chest wall pain on admission. 

**Figure 2:**
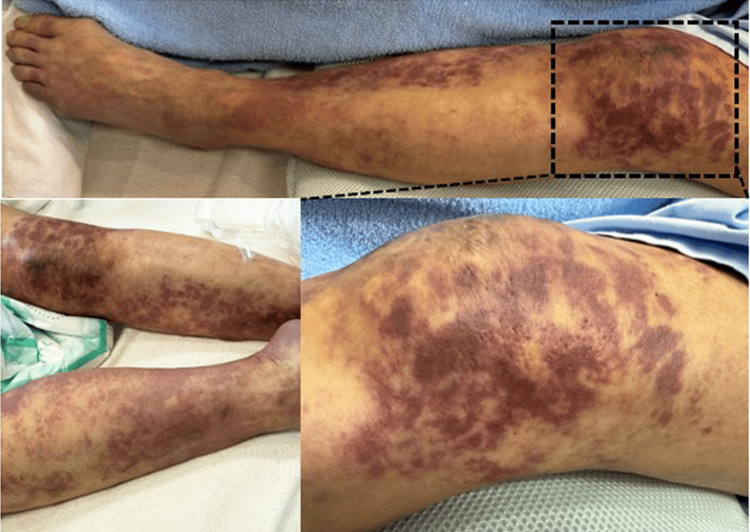
Purpura fulminans in the bilateral lower extremities. Purpura fulminans appeared approximately 10 hours after the admission.

Ten hours after the admission, the patient suddenly fell into shock status; the blood pressure (70/45 mmHg), regular heart rate (83/min), and increased respiratory rate (30/min). Saturation of percutaneous oxygen was 87% at room air. Body temperature was 36.8 ℃ and consciousness level disturbance was absent. Multiple irregular areas of dark purple cutaneous bleeding were seen on the bilateral lower extremities (Figure 2) and on the trunk including the left lateral chest wall; these findings were consistent with those of purpura fulminans (PF) [7-9].

**Figure 3:**
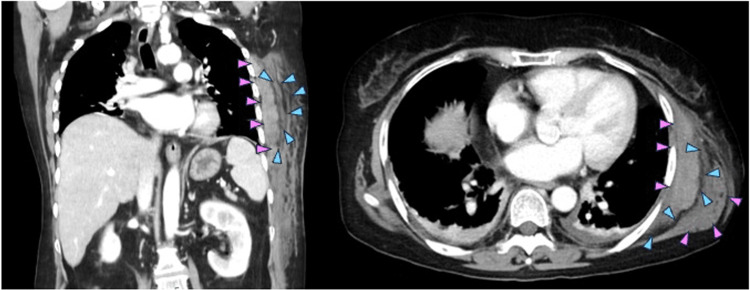
Contrast-enhanced computed tomography of the left lateral chest wall. Contrast-enhanced computed tomography revealed a subcutaneous mass in the left lateral chest wall. This mass was characterized by fluid collection along with fascia of left serratus anterior (blue arrowheads) and swelling of left serratus anterior (pink arrowheads).

Such shock status was accompanied by a marked elevation of serum levels of inflammatory mediators including CRP, procalcitonin, and interleukin-6 (IL-6, Table 1 and Figure 1). At this time point, complete blood cell counts showed mild thrombocytopenia with normal red blood cell counts and WBC. Prothrombin time and activated partial thromboplastin time were normal. Serum levels of D-dimer and fibrinogen were elevated. A marked elevation of serum creatine phosphokinase (CPK) with a normal level of CPK-MB isozyme was observed in the biochemical analyses in addition to mild elevation of serum hepatobiliary enzymes. Arterial blood gas analyses under nasal O_2_ inhalation (2L/min) were as follows; pH 7.437, PaO_2_ 52.0 mmHg, PaCO_2_ 29.7 mmHg, Base excess -4.6 mmol/L (normal range; -2-+2). Given that PF is often associated with severe acute sepsis caused by *Streptococcus* species [7-9], these laboratory findings accompanied by shock status led us to suspect NF, also known as flesh-eating disease [1-3]. Contrast-enhanced computed tomography (CT) was performed to verify the diagnosis (Figure 3). 

Contrast-enhanced CT revealed a subcutaneous mass in the lateral chest wall. This mass was characterized by fluid collection along with fascia of left serratus anterior (blue arrowheads) and swelling of left serratus anterior (pink arrowheads). These findings were fully consistent with those of NF of serratus anterior [10,11]. 

The patient received surgical debridement immediately after the diagnosis of NF together with the intravenous administration of meropenem (2.0 g/day) and clindamycin (0.6 g/day). Despite surgical incision and drainage of the left serratus anterior, serum levels of CRP, procalcitonin, CPK, and D-dimer further increased 24 hours after the admission (Table 1 and Figure 1). *S. pyrogens *was detected by culture using the drainage fluid. The present case was finally diagnosed with NF of the serratus anterior due to GAS infection. During her stay in the intensive care unit, she was on a ventilator and treated with continuous hemodiafiltration in addition to administration of catecholamines. These intensive treatments failed to rescue her from death due to multiple organ failure and septic shock. She died 11 days after the onset of symptoms.

## Discussion

The patient treated with IFX and PSL for UC and RA was diagnosed with NF due to GAS infection. Typical cases with GAS-associated NF display very rapid progression into shock status and multiple organ failure [1,2]. The patient fell into shock status and multiple organ failure from the stable condition within 10 hours. Vascular coagulopathy and cytokine storms have been considered to underlie the pathogenesis of GAS-associated NF [1,2]. PF due to thrombotic occlusion of small or middle-sized vessels as well as elevation of serum D-dimer levels was observed in the present case. Furthermore, a marked elevation of serum IL-6 levels was seen. Thus, it is clear that both vascular coagulopathy and cytokine storms are involved in the development of lethal NF in the present case. Therefore, the present case treated with IFX and PSL manifested prototypical clinical courses and pathological findings, both of which were indistinguishable from those without immunosuppressants. Although several case reports as to GAS-associated NF in patients treated with biologics and/or steroids are available, no studies have reported time kinetics of coagulation and inflammatory markers in relation to clinical manifestations by comparing these parameters with patients without immunosuppressants [12-16]. Given the potent inhibitory effects on proinflammatory cytokine responses by biologics and/or steroids, one might expect that progression of NF is slower in patients treated with these immunosuppressants than in healthy subjects. However, previous case studies have not addressed this issue. In this study, we provided laboratory data linked to clinical findings in immunosuppressive conditions and found that NF progresses very rapidly even under the treatment with immunosuppressants as seen in patients without immunosuppressants. Thus, data obtained from the present case are valuable in that appearance of PF and remarkable elevation of coagulation (D-dimer) and inflammatory markers (CRP, procalcitonin) were parallel to the severity of NF even in the immunosuppressive conditions.

GAS is the most common causative bacteria of NF [1,2]. As for the pathogenesis of NF, recent studies have successfully elucidated molecular mechanisms underlying invasive GAS infection. GAS expresses a variety of virulence factors that disrupt host innate and adaptive immunity [1,2]. These virulence factors include M protein, streptococcal pyrogenic exotoxin B (SpeB), streptolysin O (SLO), SLS, S. pyrogens cell envelope proteinase, NAD glycohydrolase (NADase), and DNAase. Attachment of GAS to the epithelial cells is mediated by M protein expressed by GAS and then SpeB, SLO, SLS, and NADase facilitate translocation of GAS into the deeper underlying tissues through disruption of epithelial barrier integrity [1,2]. After translocation into the deep tissues, most of these virulence factors enable GAS to evade host immune responses through inhibition of IL-1$\beta$, IL-8, neutrophil extracellular traps, C5a, and LL-37. In parallel to deep tissue invasion, vascular coagulopathy develops through cooperative action between SpeB, SLO, and M protein [1, 2]. Finally, it is worth noting that superantigens derived from GAS induce cytokine storms through crosslinking of major histocompatibility complex class II molecules on macrophages and dendritic cells. Thus, GAS is a well-equipped microorganism with the ability to invade the deep tissues and then promote lethal vascular coagulopathy and cytokine storms [1,2]. Importantly, the present case manifested both coagulopathy and cytokine storms as evidenced by the appearance of PF and marked elevation of IL-6. 

Although NF preferentially targets the lower and upper extremities, this condition can affect any site [1,2]. Trauma or injury proceeds in approximately 50% of patients with GAS-associated NF [1,2]. GAS can also enter the body through pharyngeal mucosa with impaired barrier function. The present case had no history of antecedent trauma or injury before the symptom onset. No history of trauma or injury led us to speculate that treatment with IFX predisposed the present case to be susceptible to GAS infection. In fact, isolated GAS-associated NF cases were reported in patients treated with TNF-$\alpha$-blockers [12,14,16]. However, no reports have provided evidence that TNF-$\alpha$--blockers increase the risk of GAS-associated NF [8]. Having said that, we cannot completely exclude the possibility that additive immunosuppressive effects by IFX and PSL were involved in the colonization and overgrowth of GAS leading to the development of NF in the present case. In line with this idea, Hamashige et al. reported a case in which NF developed in an RA patient co-treated with IFX and PSL [13]. Whether biologic therapy increases the vulnerability to invasive GAS infection requires further epidemiological and clinicopathological studies.

## Conclusions

GAS-associated NF is a serious infection with high mortality. Clinicopathological features of this disorder have been poorly understood in the presence of autoimmunity treated with immunosuppressants. Here we present time kinetics findings of clinical symptoms and laboratory data of GAS-associated NF in a patient treated with IFX and PSL for UC and RA. Accumulation of immunosuppressant-treated cases with GAS-associated NF is absolutely required to establish clinicopathological features in immunocompromised hosts. The present case highlights the need to bear in mind the possibility of GAS-associated NF upon encountering PF accompanied by shock status in a patient treated with immunosuppressive agents, including biologics.

## References

[1] Olsen RJ, Musser JM (2010). Molecular pathogenesis of necrotizing fasciitis. Annu Rev Pathol.

[2] Brouwer S, Rivera-Hernandez T, Curren BF (2023). Pathogenesis, epidemiology and control of Group A Streptococcus infection. Nat Rev Microbiol.

[3] Lappin E, Ferguson AJ (2009). Gram-positive toxic shock syndromes. Lancet Infect Dis.

[4] Bagcchi S (2023). Surge of invasive Group A streptococcus disease. Lancet Infect Dis.

[5] Abo YN, Oliver J, McMinn A (2023). Increase in invasive group A streptococcal disease among Australian children coinciding with northern hemisphere surges. Lancet Reg Health West Pac.

[6] Nygaard U, Hartling UB, Munkstrup C (2024). Invasive group A streptococcal infections in children and adolescents in Denmark during 2022-23 compared with 2016-17 to 2021-22: a nationwide, multicentre, population-based cohort study. Lancet Child Adolesc Health.

[7] Colling ME, Bendapudi PK (2018). Purpura Fulminans: mechanism and management of dysregulated hemostasis. Transfus Med Rev.

[8] Pulido-Pérez A, Bergón-Sendín M, Suárez-Fernández R, Muñoz-Martín P, Bouza E (2021). Skin and sepsis: contribution of dermatology to a rapid diagnosis. Infection.

[9] Chalmers E, Cooper P, Forman K (2011). Purpura fulminans: recognition, diagnosis and management. Arch Dis Child.

[10] Bruls RJ, Kwee RM (2021). CT in necrotizing soft tissue infection: diagnostic criteria and comparison with LRINEC score. Eur Radiol.

[11] Kwee RM, Kwee TC (2022). Diagnostic performance of MRI and CT in diagnosing necrotizing soft tissue infection: a systematic review. Skeletal Radiol.

[12] Chan AT, Cleeve V, Daymond TJ (2002). Necrotising fasciitis in a patient receiving infliximab for rheumatoid arthritis. Postgrad Med J.

[13] Hamashige J, Umemura H, Asagoe K (2018). Necrotizing fasciitis following minor skin surgery in a patient receiving treatment with infliximab and prednisolone. Eur J Dermatol.

[14] Renaud C, Ovetchkine P, Bortolozzi P, Saint-Cyr C, Tapiero B (2011). Fatal group A Streptococcus purpura fulminans in a child receiving TNF-α blocker. Eur J Pediatr.

[15] van de Sande MG, van Slobbe-Bijlsma ER (2012). Necrotizing fasciitis in a rheumatoid arthritis patient treated with tocilizumab. Rheumatology (Oxford).

[16] Choi KH, Yoo WH (2009). Necrotizing fasciitis in a patient treated with etanercept for dermatomyositis. Rheumatol Int.

